# Testing and adapting dietary habits and food security questions for a national nutrition survey using cognitive interviews and expert consultation

**DOI:** 10.1017/S1368980025101195

**Published:** 2025-10-06

**Authors:** Berit Follong, Caitlin Haliburton, Jacqueline Grey, Maria Maiquez, Sally Mackay, Lisa Te Morenga, Cliona Ni Mhurchu

**Affiliations:** 1 National Institute for Health Innovation, School of Population Health, University of Auckland, Auckland 1023, New Zealand; 2 Epidemiology and Biostatistics, School of Population Health, University of Auckland, Auckland 1023, New Zealand; 3 Centre for Translational Health Research: Informing Policy and Practice (TRANSFORM), University of Auckland, Auckland 1023, New Zealand; 4 Research Centre for Hauora and Health, Massey University, Wellington 6140, New Zealand

**Keywords:** Nutrition, Survey design, Cognitive interviewing methods, Dietary habits, Food security

## Abstract

**Objective:**

To cognitively test questions for inclusion in a national nutrition survey, ensuring the questions are interpreted as intended, and to inform further improvements.

**Design:**

A draft nutrition survey questionnaire was developed based on existing questionnaires and expert input. Twelve questions on dietary habits and food security were selected for cognitive testing as these were newly developed, amended from existing questions or identified to no longer reflect the current food environment or concepts. Cognitive interviews were conducted using both think-aloud and probing techniques to capture respondents’ thought processes used to arrive at an answer. Interviews were audio-recorded and transcribed verbatim. Qualitative data were analysed for recurring patterns and unique discoveries across the survey questions.

**Setting:**

New Zealand.

**Participants:**

Sixty-eight participants aged 11 years and older, representing diverse socio-demographics including gender, ethnicity and education level.

**Results:**

Three main cognitive challenges were identified: (1) interpreting ambiguous terms, (2) understanding dietary or technical terms and (3) following complex or unclear instructions. Questions were refined based on the study findings and further advice from experts in nutrition and survey design to enhance participant understanding and accuracy.

**Conclusions:**

The cognitive testing findings and expert input led to the refinement and potential improvement of selected questions for inclusion in a national nutrition survey. Changes included simplified terminology, clearer instructions, improved examples and better question order. Our methodological approach and findings may be valuable for those designing similar questions for dietary surveys.

Accurate and contemporary data on dietary intakes are essential for developing and evaluating evidence-based policies and programmes to improve nutrition, reduce obesity, address food insecurity and ensure food safety. In New Zealand (NZ), population diets have typically been monitored through national nutrition surveys that collect data on food and nutrient intake to assess the nutritional status of the population^(1)^. However, a national nutrition survey has not been undertaken in NZ since 2002 for children (aged 5–14 years)^(2)^ and 2008/2009 for adults (aged 15 years and over)^(3)^. Thus, a national nutrition survey is warranted to collect up-to-date population-level dietary intake data to effectively address current nutritional challenges and guide future policy initiatives.

In 2021, two NZ government agencies (the Ministry of Health and the Ministry for Primary Industries) embarked on plans for a future national nutrition survey, including the design of suitable dietary assessment questionnaires^(4)^. As NZ’s food environment and population have changed significantly since the previous national nutrition surveys, some existing survey questions were deemed likely to be outdated and potentially unsuitable for assessing contemporary dietary behaviours and household food security. For instance, NZ’s Dietary Habits Questionnaire was developed for the 2008/2009 NZ Adult Nutrition Survey and subsequently updated and cognitively tested for the NZ Health Survey in 2018/2019. The NZ Food Security Questionnaire, designed to capture a household’s access to nutritionally adequate, safe and culturally appropriate foods, was developed for the 1997 National Nutrition Survey and has not been updated since^(5)^. In 2022, a thorough review of national and international dietary habits and food security questionnaires was undertaken to identify relevant questions to assess contemporary nutrition issues^(6)^. This process led to the development of a draft dietary assessment questionnaire including new and amended questions, which had not yet been tested with the NZ population. Therefore, these questions required pretesting to explore how they were interpreted and whether further adjustments were needed.

To achieve this, cognitive testing methods were employed. Cognitive testing has been commonly used to design, evaluate and refine survey instruments^(7)^. It is used to gain insight into the cognitive processes used by respondents when answering survey questions with the goal of identifying problems with comprehension, recall and decision and answering processes^(8)^. Cognitive interviews can help identify if such issues occur, the source of the error and how a question can be improved to achieve its purpose^(9)^. This study used cognitive interviewing techniques to pretest dietary habits and food security questions in a sample of NZ children and adults from various ethnic and socio-economic backgrounds, with the aim of understanding if the questions were interpreted as intended. In addition, expert consultation was used subsequently to inform wording changes needed to improve participants’ comprehension of the questions.

## Methods

### Study design

Cognitive interviews were used to investigate respondents’ understanding of a selection of dietary habits and food security questions. Participants were asked to verbally express their thoughts and interpretations of questions that were either new, adapted or deemed unlikely to apply to today’s food environment. Findings were used to identify problematic aspects of the questions (e.g. difficult terminology, unhelpful examples or confusing instructions) to guide revision and subsequently improve the clarity and accuracy of those questions.

### Selection of questions for cognitive testing

**Fig. 1:**
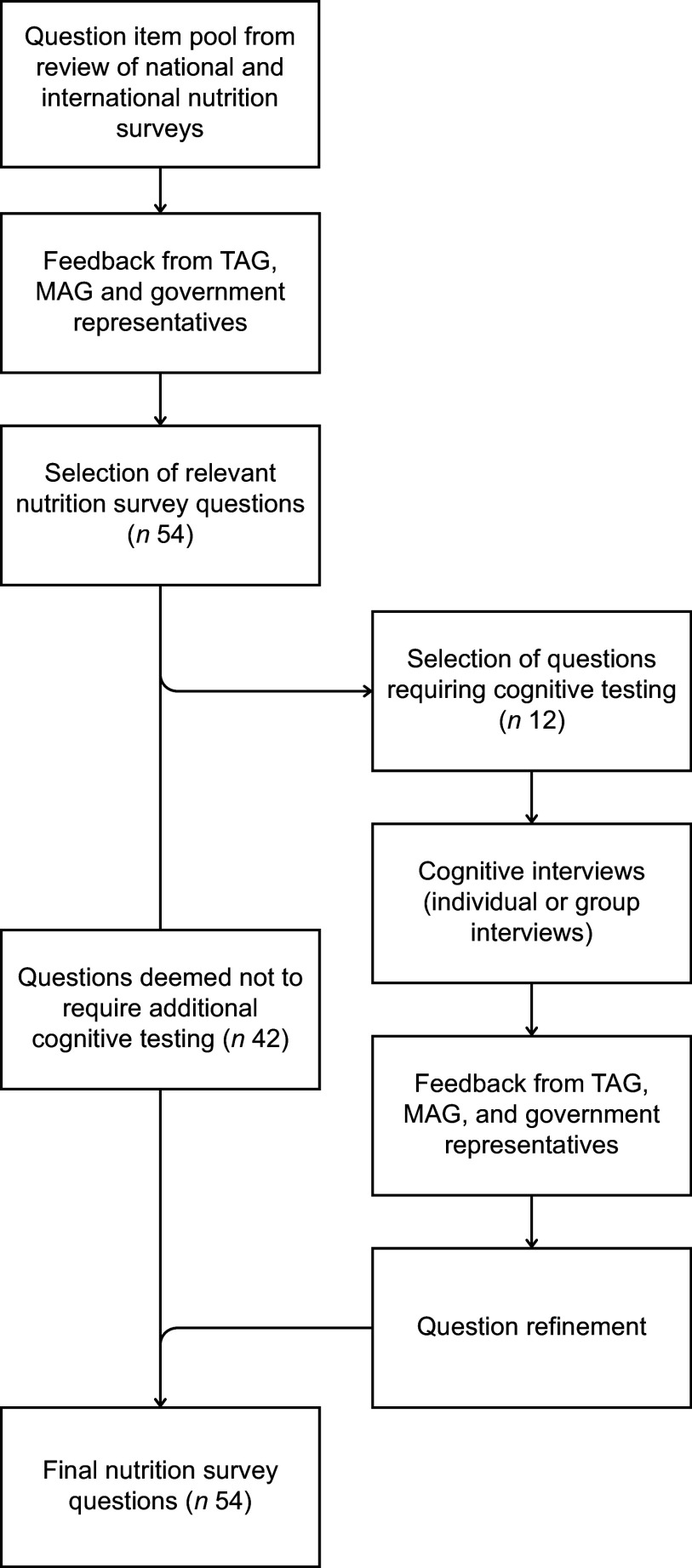
Nutrition survey question selection process and cognitive testing approach. TAG, Technical Advisory Group; MAG, Māori Advisory Group.

Work was undertaken to determine appropriate questions to include in a future NZ nutrition survey (further detailed elsewhere)^(6)^. This selection process was guided by a Technical Advisory Group (consisting of experts in human nutrition, survey design and dietary assessment), a Māori Advisory Group (including experts in Māori health and nutrition, dietary assessment and food security) and representatives from government agencies (contract funders). Together, these experts ensured the questions were appropriate for a national nutrition survey and relevant to NZ’s indigenous population (Māori). Dietary habits and food security questions from previous NZ and international questionnaires were presented to these experts, who were asked to determine if the question should be included in the future nutrition survey (i.e. suit the purpose of a national survey), to suggest any revisions (e.g. wording, order, instructions) and to propose additional questions (e.g. to cover contemporary dietary issues and current priorities). Out of the total question item pool identified, fifty-four questions were selected for inclusion in a nutrition survey. A further twelve questions required cognitive testing as they were newly developed (*n* 3), amended from the original (*n* 5) or identified to no longer be reflective of the current food environment or concepts (*n* 4). The latter was particularly relevant to the food security questions, which were developed over 25 years ago. In recent times, the concept of food security has evolved and may therefore not be accurately captured by the previously used questions. Online supplementary material, Supplemental Table 1 summarises the original questionnaire items, the questions tested in the cognitive interview (if amended) and a rationale for their inclusion and/or amendments. An illustration of the question selection process and cognitive testing approach is presented in Fig. 1.

### Study participants, recruitment and informed consent

Eligible study participants were (1) aged 11 years or older, (2) not working in or with a qualification in the field of nutrition and (3) willing to participate in a one-on-one or group interview. Although the nutrition survey will include participants aged two years and older, the recruitment age of 11 years was chosen to assess the cognitive abilities of participants who would complete the survey themselves. Parents of younger children (aged 2–10 years) complete the survey on behalf of their children, meaning that cognitive testing was not necessary for those aged under 11 years. Purposive sampling was used to recruit participants from various ethnic and educational backgrounds to ensure the questions were tested with a sample likely to include diverse perspectives and cognitive abilities. A minimum sample size of 56 was targeted in an effort to reflect NZ’s four major ethnic groups (Māori, Pacific, Asian and NZ European/Other (NZEO))^(10)^ and three key age categories (11–17 years, 18–64 years and 65+ years). A minimum of four participants per age group within each ethnic group was targeted, resulting in a base sample of forty-eight participants. A higher recruitment target was set for the Asian group to account for the broader ethnic diversity within this category. This sampling strategy was designed to capture potential differences in the interpretation of words and concepts, cognitive processing of questions and cultural eating practices^(11–13)^.

Participants were recruited using two methods, with different approaches used for specific ethnic groups. Drawing on the researchers’ long-standing networks and connections, Pacific and Asian participants were recruited through community organisations: The Fono and The Asian Network (TANI), respectively. Māori and NZEO participants were recruited through advertisements posted on local community social media groups (e.g. Facebook). People who expressed interest in the study were provided information in the form of either a child or adult participant information sheet, depending on their age. Once they agreed to participate, participants were scheduled for cognitive interviews. Adult participants were required to sign a consent form, while children provided their assent alongside parental or caregiver consent prior to taking part in the study.

### Data collection

Options for different settings for the cognitive interviews (e.g. individual *v*. group; in-person *v*. online) were provided. When scheduling the cognitive interviews, participants were given the option of participating in either a one-on-one or group interview. The procedures for both formats were consistent, other than some minor differences detailed below. Those who opted for a one-on-one interview could complete the interview in person or using an online video-calling platform (Zoom), while group interviews were conducted exclusively in person. All in-person interviews were held in participants’ homes or a community hall. Participants recruited by the community organisation partners participated in interviews that were facilitated by a trained member of that organisation, with a member(s) of the research team present where appropriate. At the start of the interview and in the consent form, participants were made aware that confidentiality could not be fully guaranteed due to the group setting and possible facilitation of interviews by a local community organisation. Participants recruited directly through local community social media groups participated in interviews facilitated by a member of the research team.

At the start of the interviews, socio-demographic information was collected for each participant. Socio-demographic data included age group (11–17; 18–64; 65+ years old), gender (male; female; other), ethnicity (one or more of the following options: Māori; Samoan; Cook Island Māori; Tongan; Niuean; Chinese; Indian; NZ European; other), education level (none; primary/intermediate school; secondary school; diploma, certificate or trade; undergraduate degree; postgraduate degree; other) and financial security (not at all – I do not have enough money to meet my basic living costs; borderline – I am just getting by financially; secure – I have money left over at the end of the month)^(14)^.

Eight dietary habits questions and four food security questions were cognitively tested. These twelve questions covered fruit and bread intake, exclusion of specific food groups, salt use, school food programmes, food procurement, dietary supplement use, access to basic foods, reliance on different sources of food assistance and food preparation and storage facilities. The questions were available in English only, as the eventual NZ nutrition survey, similar to other national health surveys, will not be translated into other languages. Some questions were deemed only appropriate for respondents aged 18 years or older, and therefore, participants aged 11–17 years were asked six out of twelve questions. Questions not asked of younger participants included the four food security questions, the food procurement question and another on food preparation and storage facilities. These questions required participants to answer on behalf of their household, with adult respondents considered more appropriate for this task given their role within the household and greater involvement in food preparation or purchasing activities. Participants were presented with showcards^(15)^, including the questions, answer options and images where appropriate (e.g. examples of the foods that the question referred to or typical serving sizes – see online supplementary material, Supplemental File 1). All questions and answer options tested are listed in online supplementary material, Supplemental Table 1. Although answers to questions were recorded, the focus of this study was participants’ interpretation of the questions and how they came to an answer. Participants’ answers were therefore not analysed.

The interview process was outlined to participants at the beginning of the session. A combination of the think-aloud and the verbal probing techniques was used to elicit responses and gain insight into participants’ cognitive processes^(8,9)^. While participants in individual interviews read aloud each question and then answered them, questions were read aloud by the interviewer in group interviews, where participants then took turns in answering if comfortable doing so. Participants were reminded to verbalise their thoughts as they worked through answering the question. Following each question, the participants were asked a series of additional probing questions (both pre-defined and spontaneous) if they were unable to adequately articulate their thought processes. Examples of probing questions used include ‘You picked [answer], how did you come up with that?’, ‘In your own words, what do you think the question is asking?’ and ‘How would you word this differently?’. In addition, participants were asked to provide feedback on the showcards (e.g. ‘What are your thoughts on this showcard?’).

Each session lasted 0·5–1·5 h and was audio-recorded. Participants received a gift voucher for their participation in the study. All interviews were conducted between September and December 2022.

#### Transcription, analysis and question refinement

Socio-demographic data were summarised using descriptive statistics including percentages for categorical variables and mean (sd) for continuous variables (Excel, version 2308, Microsoft Corporation). All audio recordings were transcribed verbatim by one of five members of the research team. One researcher (BF) reviewed all interview transcripts, and participants’ responses were grouped by test question. The grouped responses were summarised (BF) using short descriptive codes that captured key ideas or meanings. Further inductive analysis was conducted by two researchers (BF and CH) who reviewed, refined and grouped the initial codes^(13)^. Themes (i.e. issues that occurred repeatedly) were identified by comparison and grouping of the coded data for each question across all interviews. In addition, responses that were particularly insightful, regardless of their frequency (i.e. discoveries), were captured^(16)^. Coding ambiguities and findings were reviewed and discussed in consultation with a third researcher (SM) to ensure consistency between coders. To illustrate and support each theme, relevant quotes from participants were selected. Findings were not compared by age or ethnic group because the numbers in each subgroup were small. Following the analysis, findings were presented to and discussed with government representatives, the Technical Advisory Group and the Māori Advisory Group to decide on improvements to questions.

## Results

### Study participants

Sixty-eight study participants took part in either group interviews (*n* 16 interviews, each involving between two and fifteen participants) or in one-on-one interviews (*n* 14). Socio-demographic characteristics are outlined in Table 1 for the total sample and by age group. The sample was predominantly female (*n* 48) with approximately equal representation by key ethnic groups, education level and financial security.

**Table 1.** Socio-demographic characteristics of study participants

Socio-demographics	Total (*n* 68)		Age groups					
	*n*	%	11–17 years (*n* 22)		18–64 years (*n* 26)		65+ years (*n* 20)	
			*n*	%	*n*	%	*n*	%
Gender								
Male	20	29	9	41	6	23	5	25
Female	48	71	13	59	20	77	15	75
Ethnicity								
Māori	13	19	5	23	4	15	4	20
Pacific	21	31	6	27	11	43	4	20
Asian	22	32	7	32	7	27	8	40
New Zealand European or other	12	18	4	18	4	15	4	20
Education level (highest qualification completed)								
Primary/intermediate school	16	24	15	68			1	5
Secondary school	19	28	7	32	8	31	4	20
Diploma, certification or trade	6	9			4	15	2	10
Undergraduate degree	12	18			8	31	4	20
Postgraduate degree	14	20			6	23	8	40
Decline to answer	1	1					1	5
Financial security^*^								
Not at all – I do not have enough money to meet my basic living costs	1	2					1	5
Borderline – I am just getting by financially	16	45			11	42	5	25
Secure – I have money left over at the end of the month	24	52			13	50	11	55
Decline to answer	5	11			2	8	3	15

* Excluding participants aged < 18 years.

Although the questions tested were generally well understood, some aspects were identified that impacted participants’ interpretation of questions and subsequently their answers. These were (1) interpretation of ambiguous terms, (2) poor understanding of dietary or technical terms and (3) difficulty following complex or unclear instructions. Below, each theme is described more fully, and examples are provided. Table 2 details the questions presented for cognitive testing, the issues identified from the thematic analysis, the refinements made based on the findings and further discussion with expert advisors and the resulting final questions.

**Table 2.** Summary of identified issues, modifications and revised questions

#	Question for cognitive testing	Issues identified	Modifications	Revised question^*^
1	On average, how many servings of fruit do you eat per day? Please include all fresh, frozen, canned and stewed fruit. Do not include fruit juice or dried fruit. A ‘serving’ = 1 medium piece or 2 small pieces of fruit or ½ cup of stewed fruit. For example, 1 apple + 2 small apricots = 2 servings. I don’t eat fruit Less than 1 serving per day 1 serving per day 2 servings per day 3 servings per day 4 or more servings per day Don’t know Refused	**Poor understanding of dietary or technical terms** Difficulty with understanding the concept of a ‘serving’. Some participants think of a serving as one ‘piece’ of fruit.	An additional example of a serving was included, and examples were changed to less seasonal fruits.	How many **servings** of fruit do you **usually** eat per day? Please include all fresh, frozen, canned, stewed fruit and fruit added to smoothies. Do not include fruit juice or dried fruit. A **‘serving’** of fruit = 1 medium piece or 2 small pieces of fruit or 1 cup of fruit (canned, frozen or stewed). For example, 1 apple + 2 small mandarins = 2 servings. I don’t eat fruit Less than 1 serving per day 1 serving per day 2 servings per day 3 servings per day 4 or more servings per day Don’t know Prefer not to say
		**Complex or unclear instructions** Difficulty coming up with an ‘average’. Not including fruit added to smoothies in their response.	Changed ‘on average’ to ‘usually’. Instructions were included to prompt for fruit added to smoothies.	
2	On average, how many servings of bread do you eat per day? A ‘serving’ = 1 slice of bread (40 g) or ½ medium bread roll (40 g) or ½ naan bread (35 g). Other examples: ½ English muffin or 1 pita bread. I don’t eat bread Less than 1 serving per day 1 serving per day 2 servings per day 3 servings per day 4 servings per day 5 servings per day 6 or more servings per day Don’t know Prefer not to say	**Interpretation of ambiguous terms** Participants included other ‘carbohydrate’ sources such as noodles and rice when coming up with an answer.	Bolded the word ‘bread’ and included an interviewer note to prompt participants to not include all high-carbohydrate-containing foods.	How many **servings** of **bread** do you usually eat per day? A **‘serving’** of bread = 1 slice of bread or ½ medium bread roll or ½ naan bread. Other examples: ½ English muffin or 1 pita bread or ½ bagel. I don’t eat bread Less than 1 serving per day 1–2 servings per day 3–4 servings per day 5–6 servings per day 7 or more servings per day Don’t know Prefer not to say
		**Complex or unclear instructions** Confusion about which ‘bread’ products should and shouldn’t be considered here. Difficulty coming up with an ‘average’.	Given the complexities of folate fortification in bread in New Zealand, to ensure accuracy, a complete list of ‘breads’ that should be included in this question should be supplied during the interview (available to the interviewer). Changed ‘on average’ to ‘usually’.	
		**Poor understanding of dietary or technical terms** Difficulty with understanding the concept of a ‘serving’ as serving sizes presented were different from how the food items are usually consumed (e.g. a whole bread roll instead of half). Most understood one slice of bread was one serving.	Serving size images were not changed to reflect common consumption quantities to maintain consistency with other serving size questions. The gram weights for the serving size examples were removed to avoid confusion.	
3	Do you eat any of the following foods? Select all that apply. Red meat (e.g. beef, pork, mutton, lamb, goat, venison) Chicken or poultry (e.g. turkey, duck) Fish or other seafood Eggs Dairy products (e.g. milk, cheese) Gluten sources (e.g. wheat, barley) Nuts Don’t know Prefer not to say	**Complex or unclear instructions** Confusion about which answer to select if participants do not eat all of the foods listed as examples. Some participants left answer options unticked if they eat the food only occasionally, and others wanted to be able to answer ‘sometimes’ or ‘not very often’. Some participants only considered whole nuts and not foods made with nuts like nut butter, nut bars, etc.	Changed question wording back to the original question as in the New Zealand Health Survey 2018–2019 (i.e. completely exclude) to reduce ambiguity. Removed ‘nuts’ as an answer option because knowing if consumers eat nuts is not of policy relevance.	Do you completely exclude any of the following foods? Select all that apply. Red meat (e.g. beef, pork, mutton, lamb, goat, venison) Poultry (e.g. chicken, turkey, duck) Fish or other seafood Eggs Dairy products (e.g. milk, cheese) Gluten (e.g. bread, pasta) Don’t know Prefer not to say
		**Poor understanding of dietary or technical terms** The term ‘gluten sources’ was not well understood.	It was assumed that by changing the question to ask about those foods that are completely excluded, it would be easier to know whether to tick this answer or not. Changed the answer option to ‘gluten’ and provided clearer examples of gluten-containing foods (e.g. bread).	
4	How often do you/does the person who prepares your food add salt when you/they are cooking or preparing food? Never Rarely Sometimes Regularly Always Don’t know Prefer not to say	**Complex or unclear instructions** Participants sometimes included salt added at the table when answering this question. Few participants mentioned that salty sauces are often used in place of salt and wondered whether they should be considered in their answer to this question.	Both questions will be displayed to the participant at the same time so that they are aware that the two behaviours are asked about separately. Used the wording from the WHO STEPS questions to incorporate salty sauces.	a) How often is salt, salty seasoning or a salty sauce added in cooking or preparing foods in your household? Always Often Sometimes Rarely Never Don’t know Prefer not to say b) How often do you add salt or a salty sauce such as soy sauce to your food right before you eat it or as you are eating it? Always Often Sometimes Rarely Never Don’t know Prefer not to say
5	In the past 12 months, have you used any of the following food programmes? Please select all that apply. Breakfast programme Fruit in schools Milk in schools Free and Healthy School Lunch programme (e.g. Ka Ora, Ka Ako) Charitable food programme (e.g. KidsCan, Eat My Lunch) Other, please specify (leave blank if unknown) I don’t receive food and/or drinks from food programmes Don’t know Prefer not to say	**Complex or unclear instructions** Some misunderstanding about whether the question is asking about school or early learning services.	Included the word ‘school’ in the question and added another example. Answer options were amended to reflect changes in the availability of school food programmes.	In the past 12 months, have you used any of the following school food programmes? Please select all that apply. Breakfast programme (e.g. KickStart) Fruit in Schools programme Free and Healthy School Lunch programme (e.g. Ka Ora, Ka Ako) Charitable food programme (e.g. KidsCan, Eat My Lunch) Other, please specify (leave blank if unknown) I don’t receive food and/or drinks from food programmes Don’t know Prefer not to say
6^†^	In the past 12 months, has your household eaten foods from any of the following sources? Include both foods that your household has gathered/collected and those that were given to you. Please select all that apply. Hunting Fishing/diving Home kill Foraging (fruit, vegetables, nuts, herbs, mushrooms) Your (community) garden Collecting fresh eggs Milking cows/sheep/goats Other, please specify No, I buy all my food *Followed by a frequency input for any selected foods.* How often does your household eat foods from [food source]? Most days Weekly Monthly 3–4 times per year 1–2 times per year Don’t know Prefer not to say	**Poor understanding of dietary or technical terms** The term ‘foraging’ was not well understood.	Changed the examples to foods that are likely to be foraged (e.g. puha, watercress, berries, mushrooms).	In the past 12 months, has your household eaten foods from any of the following sources? Include both foods that your household has gathered/collected and those that were given to you (but not paid for). Please select all that apply. Hunting Fishing/diving/collecting shellfish Home kill Foraging (e.g. puha, watercress, berries, mushrooms) A garden/māra/community garden (do not include herbs) Collecting fresh eggs Milking cows/sheep/goats No, I buy all my food Don’t know Prefer not to say *Followed by a frequency input for any selected foods.* In the past 12 months, how often has your household had/eaten [food source]? Most days Weekly Fortnightly Monthly Every 2 months 1–5 times per year Don’t know Prefer not to say
		**Complex or unclear instructions** The ‘your (community) garden’ answer option was unclear as participants did not know what type of gardens needed to be considered. Several participants reported getting herbs from their garden, which may have significantly inflated their answers. The ‘other, please specify’ answer option did not make sense in this context. The frequency question was perceived as difficult to answer as consumption of foods from these sources is irregular or seasonal. Answer options did not reflect this.	Changed the answer option to ‘a garden/māra/community garden’. Provided instructions not to include herbs. Removed this answer option. Answer options to the frequency question were modified.	
		**Interpretation of ambiguous terms** Confusion around how to answer this question when the ‘household’ is a flat or shared house, but food is not shared.	The ‘family unit’^‡^ concept from the US National Health and Nutrition Examination Survey (NHANES) will be used for all relevant questions.	
7	Have you taken any dietary supplements in the past month? Include any prescription and over-the-counter supplements. Yes No Don’t know Prefer not to say	**Poor understanding of dietary or technical terms** The term ‘dietary supplements’ was not well understood.	Clarified what is considered a dietary supplement by rewording the question to include ‘have taken any vitamins, minerals, herbals or other dietary supplements’	Have you taken any vitamins, minerals, herbals or other dietary supplements in the past month? Include any prescription supplements. Dietary supplements are defined as anything you consider to be a supplement to your diet. Dietary supplements are not intended to replace an entire food, meal or diet. These are taken orally (e.g. as a capsule, tablet, liquid, powder) or given by injection. *[Interviewer instructions: If asked, please do not count protein powders, meal replacements and sports supplements.]* Yes No Don’t know Prefer not to say
		**Interpretation of ambiguous terms** The term ‘over the counter’ was not well understood.	Removed ‘over the counter’ to mitigate issue and knowing that dietary supplements are also commonly purchased online.	
		**Complex or unclear instructions** Unclear whether to include protein powders, meal replacements and other sports supplements.	Included an interviewer instruction that these should not be included in the dietary supplement definition.	
8^†^	*We are interested in whether you run out of basics, like bread, potatoes, etc., because you do not have enough money. We are NOT referring to treats or special foods.* Food runs out in my/our household due to a lack of money. How often has this been true for you (or your household) over the past year? Often Sometimes Never Don’t know Refused	**Interpretation of ambiguous terms** Participants’ interpretations of ‘basics’ were varied and often considered major food groups such as meat, vegetables, fruit, etc. Bread and potatoes were not considered basics in some cultures. Confusion around how to answer this question when the ‘household’ is a flat or shared house, but food is not shared.	Examples were removed, and ‘basics’ was left open to interpretation. The ‘family unit’^‡^ concept from the US National Health and Nutrition Examination Survey will be used for all relevant questions.	*We are interested in whether you run out of basic foods because you do not have enough money. We are NOT referring to treats or special foods.* Food runs out in my/our household **due to a lack of money**. How often has this been true for you (or your household) over the past year? Often Sometimes Never Don’t know Prefer not to say
9^†^	*Some people rely on support and assistance from others for supplying their regular food, and we are interested in finding out how many people fall into this group.* I/we rely on others to provide food and/or money for food, for my/our household, when I/we don’t have enough money. How often has this been true for you (or your household) over the past year? Often Sometimes Never Don’t know Refused	**Complex or unclear instructions** Some participants described reasons other than lack of money that they had run out of food. Food security questions are too long and wordy.	Q8 will be used as a screening question and ‘due to a lack of money’ was bolded. This could help focus on the lack of money only and reduce the burden on people who have never experienced this. Questions 9–11 were combined to distinguish the different types of support or assistance clearly and to reduce the wordiness.	*[The following questions are only asked if indicated ‘often, sometimes, don’t know or prefer not to say’ to the screening question]* *Some people rely on support and assistance for supplying their regular food and we are interested in finding out how many people fall into this group.* How often has the following been true for you (or your household) over the past year? I/we rely on **whānau/family, friends or neighbours** to provide food and/or money for food, for my/our household, when I/we don’t have enough **money**. Often Sometimes Never Don’t know Prefer not to say I/we make use of **food grants or food banks** when I/we do not have enough **money** for food. Often Sometimes Never Don’t know Prefer not to say I/we receive support from a **church, marae or other community organisation** when I/we do not have enough **money** for food. Often Sometimes Never Don’t know Prefer not to say
		**Interpretation of ambiguous terms** It was unclear who ‘others’ was referring to.	Provided examples of ‘others’ (i.e. friends, family and neighbours)	
10^†^	*Also, some people have to rely on other sources of help such as food grants or food banks.* I/we make use of special food grants or food banks when I/we do not have enough money for food. How often has this been true for you (or your household) over the past year? Often Sometimes Never Don’t know Refused	**Complex or unclear instructions** Some participants described reasons other than lack of money that they used these sources of help.	See Q9.	
11^†^	*Some people have to rely on other sources of help such as their church, their marae or other community organisations.* I/we receive support from a church, marae or other community organisation when I/we do not have enough money for food. How often has this been true for you (or your household) over the past year? Often Sometimes Never Don’t know Prefer not to say	**Complex or unclear instructions** Support received from churches, marae and other community organisations was considered by participants when answering the previous two questions.	Kept this new food security question to include churches, marae and other community organisations in the list of support sources. See Q9.	
12^†^	Are your current food preparation and food storage facilities adequate to prepare food for your household? Yes No Don’t know Prefer not to say	**Complex or unclear instructions** Large variety of ‘food storage’ facilities were considered, but not many participants considered ‘food preparation’ facilities when answering.	Reworded the question to use active terms ‘store’, ‘prepare’ and ‘cook’.	I/we have good enough facilities to store, prepare and cook food for my/our household. Yes No Don’t know Prefer not to say
		**Interpretation of ambiguous terms** The term ‘adequate’ was not always well understood.	Changed to ‘good enough’.	

* Questions were refined based on cognitive interview findings and discussions with advisory group members and Ministry representatives.

† Questions were only asked to adult participants.

‡ Family unit: Everyone related to each other by blood, marriage or a marriage-like relationship including partners and foster children.

#### Interpretation of ambiguous terms

Several terms were interpreted inconsistently across participants, such as ‘basics’, ‘bread’, ‘household’, ‘others’, ‘adequate’ and ‘over the counter’. One food security question (Q8) asked participants to indicate whether they or their household ran out of basic foods due to a lack of money, with examples provided being ‘bread, potatoes, etc.’. When prompting participants to explain what type of basic foods they thought of, a wide variety of answers were given. Answers ranged from starchy types of foods, *‘bread, potatoes, rice, pasta’*, to individual ingredients used for cooking or baking, such as *‘butter, sugar, eggs, flour, milk’*, to the inclusion of almost all food categories within the dietary guidelines, like *‘fruit, vegetables, meat, fish, potatoes, bread’*. Further discussions with the expert advisors revealed that it is important to capture cultural and generational differences in the interpretation of basic foods. It was therefore decided to keep the terminology open to interpretation and to remove the specific examples.

One dietary habits question asked about the consumption of bread (Q2). Examples of bread products were listed to prompt the participants to think of those and similar foods. However, when participants tried recalling the average number of servings of bread they consume per day, they included non-bread products. Several participants confused the term ‘bread’ with ‘carbohydrates’, asking for clarification, *‘So, I know bread as carbs. So is it, this question is directed to bread or carbs?’ (Female, 18–64 years)*. While most participants talked about slices of toast they would usually have, there were examples of participants including other grain foods such as breakfast cereals, rice and noodles.

The term ‘household’ was used in multiple questions (Q6, Q8–12), and the interpretation was perceived as challenging by some. With different living arrangements come different approaches to purchasing and preparing food. Those participants who lived in shared housing said that living with others did not necessarily mean sharing their food with their housemates. They were unsure whether to interpret the term ‘household’ as everyone they live with or only those with whom they share their food expenses:

‘Uh well other people in our house go fishing, diving, they have been given home kill, uh maybe fresh eggs as well, but we have not eaten them.’ and ‘Hmm and I guess for the household again, for example, it might be one of our flatmates [housemates] might have used it [food assistance] often, but we might never use it and so how would you know what to put?’ (Female, 18–64 years)

This issue is relevant to four food security questions, a question on food preparation and food storage facilities and a household food procurement question. After in-depth discussions with our expert advisors and considering typical household composition in NZ for different ethnic groups, it was decided that a household should be defined as the ‘family unit’ concept used in the US National Health and Nutrition Examination Survey (see Table 2)^(17)^. An interviewer should clarify this concept before reading out the household questions to help participants better understand whom to consider in their answers.

Three of the selected food security questions asked about the reliance on a different source when the participant’s household ran out of food due to a lack of money (i.e. ‘others’, ‘food banks or food grants’, ‘churches, marae [Māori meeting house] or other community organisations’). For the first question, it was unclear who ‘others’ referred to, and participants interpreted it variably (Q10). As a consequence, participants’ responses included several sources that should have been included in subsequent questions. This sometimes led to confusion when reading the second and third questions, thinking they had already answered this, and they were repeated questions. For example, after reading the first question, a participant answered, *‘I think I may have only gotten a food grant like once or twice since the start the year’ (Male, 18–64 years)*, so selected ‘sometimes’ as the frequency answer, which was incorrect, given that the first question was intended to explore the use of personal sources of support like friends and family. Once prompted, they explained they did not consider friends and family in this question as they *‘never really relied on my whānau [family] to provide kai [food] if needed’ (Male, 18–64 years)*. As a result of these findings, the term ‘others’ was changed to ‘whānau/family, friends or neighbours’ to clarify what was being asked about.

#### Poor understanding of dietary or technical terms

Some participants had difficulty understanding dietary terms in questions or answer options, such as ‘serving size’, ‘gluten sources’, ‘dietary supplements’ or ‘foraging’. Two dietary habits questions asked participants to estimate the number of servings of fruit (Q1) and bread (Q2) they consumed on average over the past four weeks. The showcards included multiple visual examples of what constitutes a single serving (e.g. ‘1 medium piece’ showing an image of the palm of a hand with an apple). Often, participants noted examples of a serving that did not align with the instructions on serving sizes. While one serving of fruit could be made up of two smaller pieces (e.g. two plums) or one serving of bread could be half the size of how a product is typically consumed (e.g. half a bread roll), participants generally counted the times that they ate a food or the number of pieces they had, independent of the size of that item. One participant said, *‘I was counting one kiwifruit as one serving’ (Female, 65+ years)*, while another participant said, *‘Yeah, I would think most people would count one roll’ (Female, 18–64 years)*. The questions and showcards were subsequently amended to include more (and less seasonal-dependent) examples (and visuals) of a serving size, and gram weights were removed.

Another dietary habits question asked participants to select all the foods that they eat from a multiple options list (e.g. red meat, dairy products) (Q3). One of the answer options, ‘Gluten sources (e.g. wheat, barley)’, was poorly understood by some as they were unfamiliar with the term. To simplify, the question was reframed to ‘Do you completely exclude any of the following foods?’. The revised question should be easier to answer as only people who completely avoid gluten would answer yes. The examples of gluten sources were changed to bread and pasta to represent actual foods consumed rather than the grains, and ‘sources’ was removed.

Participants’ understanding of the term ‘foraging’ was variable (Q6). Some participants were able to provide a brief explanation of what they considered foraging to be, but others said that they did not know what the term was or thought it was similar to one of the other answer options. The examples listed for foraging (i.e. fruit, vegetables, nuts, herbs, mushrooms) were perceived as confusing as these foods could generally also be obtained through other sources such as a home or community garden. One participant was unsure, ‘*Is foraging is that, is that what, is that just picking fruit from your backyard or is it that, what that is foraging?’ (Male, 18–64 years)*. The answer examples were subsequently changed to help respondents distinguish the difference between foraging and food usually obtained from a (community) garden by being more specific to foods sourced from the wild.

#### Difficulty following complex or unclear instructions

Participants had difficulty selecting the correct answer option in instances where instructions were more complex (e.g. averaging intake of fruit over four weeks) or unclear (e.g. types of foods included or excluded in specific food groups). For questions on the number of servings of fruit and bread (Q1–2), an example calculation was given (i.e. ‘1 apple + 2 small apricots = 2 servings’). Some participants had difficulty answering, stating that their intake of these foods varied significantly throughout the week. One younger participant selected ‘I don’t know’, ‘*Because some days I just eat a random amount of fruit, so some days I have three servings and some days I have one’ (Male, 11–17 years)*. Additionally, calculating an average over the past four weeks was a complex task, which was likely amplified by difficulties related to estimating serving sizes as described earlier:

‘I am just confused about the serving, what we should put, because normally, out of thirty days of the month, we eat twenty days roti made at home. And they can be different in the size. Some people make very small, like I make very small, and thin. Some make big, and… so it is confusing to put the serving, how many servings should we put?’ (Female, 65+ years)

Participants tried to be precise in their estimation, resulting in cognitively difficult response processes. To overcome this issue, the term ‘on average’ was replaced with the term ‘usually’ in revised questions.

The food security questions, in general, were perceived as complex and wordy (Q8–11). This may explain why instructions were sometimes overlooked or unclear (i.e. the questions related to specific situations where people *rely on food support* due to the *lack of money*). For example, participants talked about situations where they had to rely on people or organisations but not due to the lack of money. The COVID-19 pandemic was a key reason for relying on food support, *‘Uh, I will say “sometimes”, but it was not due to money. It was due to not being able to go and get food because of COVID’ (Female, 18–64 years)*. The questionnaire structure for the food security questions was subsequently amended to simplify the instructions and ensure that participants answered affirmatively only when lack of money was an issue.

Two issues were identified for the question on salt added to food during preparation or cooking (Q4). A participant questioned whether salty sauces should be considered in answering the question on discretionary salt use:

‘Hmm this was not as easy as it should have been to answer. And the reason is, for myself, I never add salt to food. However, I cook Thai, so we use fish sauce, which is pretty much 50 % salt, is not it? So I have hidden salt. I do not get the white salt and add it.’ (Female, 65+ years)

Discussions with the expert advisors indicated it was important to capture the use of salty sauces, and better instructions were needed to ensure the question was interpreted as intended. The question was therefore rephrased to align with the WHO’s STEPS questions on salt use, which include salty sauces^(18)^. While the salt question specifically asked about salt added in cooking or preparing foods, there was some confusion around whether salt added at the table should be included. Several participants noted their salt use after food had been prepared: *‘I would not always shake it on. You know, like sometimes I will just leave it and just eat what that person has prepared’ (Female, 65+ years).* The full draft nutrition survey questionnaire includes two salt questions (i.e. added while preparing or cooking *v*. added at the table), the latter of which was not cognitively tested. To avoid confusion, both salt questions were displayed on one showcard for participants to be able to distinguish between the two different behaviours. No other formatting changes were made to the showcards that could potentially influence the interpretation of the questions.

## Discussion

We aimed to cognitively test several questions selected for inclusion in a national nutrition survey to inform expert discussions, which together would guide the revisions needed to improve the questions. The cognitive testing undertaken with children and adults provided useful insights into respondents’ understanding of twelve questions assessing specific dietary habits and household food security. Challenges experienced by the participants were revealed using the think-aloud technique, while additional issues, sometimes not identified by participants, were identified through probing. The findings indicated where further refinements to questions and answer options were required to improve participant comprehension. Specifically, ambiguous terms were clarified, more technical dietary terms were simplified where possible, additional examples and descriptions of foods were added, more detailed explanation was provided in some question instructions and question order was adapted to increase questionnaire logic. The variable interpretations of key concepts and instructions in the questions, as originally worded, suggest that the validity of answers would likely have been reduced, highlighting the importance of cognitive testing as part of standard survey design to improve questions and maximise accuracy and validity. The process, from question selection through cognitive testing, expert consultations and subsequent amendments to questions, was iterative in nature. The discussion of test findings with experts and government representatives was a distinctive feature of this approach, ensuring any proposed changes would still adequately measure dietary habits and food security, align with the purpose of a national nutrition survey and maintain the ability to measure change over time using previous national survey data. Government representatives also considered how amendments would inform nutrition policy or food regulation. As these dietary habits and food security questions have been used in previous NZ national surveys, our findings are relevant to future nutrition surveys and to researchers considering using these or similar questions in other dietary studies. Even for questions that have been used frequently and over a prolonged period, it cannot be assumed that such questions remain appropriate or relevant without review. This underscores the need for regular cognitive testing to ensure that nutrition survey questions continue to accurately capture dietary habits and behaviours and reflect contemporary understandings and contexts.

Findings are consistent with similar studies in other populations, which also identified issues related to a lack of clarity in question instructions and wording^(11,19,20)^. Various studies that cognitively tested either nutrition survey questionnaires or FFQ’s have reported that diet-related terms and concepts (e.g. fruits, beans, serving size, low fat, 100 % fruit juice) were misinterpreted or interpreted inconsistently^(11,20–24)^. For example, in a study by Wolfe *et al.* (2001)^(11)^, participants found the term ‘serving’ difficult to define and included a wide range of examples that they considered to be a serving. Like these studies, we subsequently changed question wording or added more examples and instructions to address the cognitive challenges identified. Unfamiliarity with the concept of servings and difficulties in estimating the average servings consumed over a specific time period have commonly been reported in previous research^(19,25)^. Even in other disciplines, such as physical activity, questions that involve determining the frequency and duration of activities are difficult^(26)^. A recent study by Drolet-Labelle *et al.* (2024)^(19)^ reported that older adults struggled to provide a response to an FFQ when having to calculate serving sizes and frequency of consumption. Changing question wording to ‘usually’ rather than ‘on average’ may help participants formulate a response, given that people tend to describe ‘typical’ or ‘normal’ patterns and behaviours when answering such questions^(26)^. Furthermore, cognitive testing of questions frequently reveals that instructions on how to answer a question may be sub-optimal^(11,19,21,24,26)^.

With the food security questions we tested, several participants said that they found them complex and wordy and thus may have overlooked specific instructions referring to times that they ran out of money. Our findings indicate that people consider other factors beyond financial constraints to significantly impact their household’s food security, with the COVID-19 pandemic and its associated restrictions limiting access to food being mentioned most frequently. Furthermore, questions were deemed somewhat repetitive, and participants expressed uncertainty around which support services to include in their responses. Similar findings have been reported in studies exploring participants’ understanding of the US Household Food Security Module^(12,27)^. For instance, college students perceived the Household Food Security Module questions as repetitive and reported non-financial reasons for having to make less healthy dietary choices (e.g. time and transportation constraints) despite the question wording focusing on financial insufficiency^(12)^. This may lead to misclassification of food security levels and, in turn, could have a major impact on policy implications. As such, several researchers have identified the need for more comprehensive food security questionnaires^(28–30)^. Food security is multifaceted and context specific, with the FAO defining it as *‘All people, at all times, have physical, social and economic access to sufficient, safe and nutritious food which meet their dietary needs and food preferences for an active and healthy life’* ^(31)^. Recently, Calloway and colleagues developed food security questions addressing additional pillars of food security (i.e. availability, utilisation and stability), which can be used alongside the standard US Household Food Security Module questionnaire^(32)^. In addition to the amendments we propose to the NZ food security questions, further research is recommended to identify new question domains relevant to the diverse NZ population to capture a broader experience of food insecurity.

This study had several strengths. It was conducted with a relatively large and diverse sample, capturing a wide range of possible perspectives, misinterpretations or understandings. Both think-aloud and probing techniques were used, followed by a detailed and rigorous analysis of the responses, to gain a comprehensive understanding of participants’ cognitive processes. No heterogeneity was observed in the findings of the group and individual interview modes, suggesting that offering both options could be a valuable approach for others. It allows for more flexible and participant-tailored methods of data collection. Expert advisors were involved in key steps of the process to consider the impact of the possible changes in question wording on the purpose and aims of a national nutrition survey. There were, however, some limitations. The generalisability of the findings of this study is limited, given the highly specific nature of the questions and the profile of the population they were tested with. The group interview format may have meant that some participants did not feel comfortable or able to respond to all questions. Despite a total sample of sixty-eight participants, when considered by age, gender and ethnic group, this resulted in small numbers in some subgroups, and detailed analysis was therefore only undertaken for the total sample. Additional cognitive issues relating to the food security questions may not have been captured, given that a relatively small proportion of study participants self-reported low financial security. Moreover, this research was conducted soon after the COVID-19 pandemic in NZ, with many participants referring to how being isolated had impacted their food security. Only a selection of questions (*n* 12) from the proposed nutrition survey were tested to minimise participant burden. Feedback showed that the omission of related questions may have led to some confusion (e.g. for the salt question). Cognitive testing typically follows an iterative approach, involving multiple rounds of testing with revisions made to the questions based on participant feedback within each round. However, we were unable to use such an approach due to time and budget constraints. Although the revisions made are likely to improve the accuracy of data collected, revised questions should be retested on a similar sample to ensure that revisions have improved respondents’ comprehension of the questions and have not introduced new areas of confusion.

### Conclusion

The cognitive testing of twelve questions proposed for inclusion in a national nutrition survey provided valuable data, which led to the refinement and potential improvement of selected questions. A key feature of this study was engaging experts and government representatives in reviewing the findings to ensure proposed question wording changes remained aligned with the nutrition survey goals. Changes included simplified terminology, clearer instructions, improved examples and a more logical question order. These findings highlight that cognitive interviewing is a useful method when designing survey questionnaires to identify any problems in understanding questions as intended and thus develop more effective survey measures. Cognitive testing of questions used in national surveys should be conducted periodically to assess participants’ ongoing understanding of questions, though for studies where a time series is important, amendments need to be carefully considered.

## Supporting information

Follong et al. supplementary material 1 Follong et al. supplementary material

Follong et al. supplementary material 2 Follong et al. supplementary material

## Supplementary material

For supplementary material accompanying this paper, visit https://doi.org/10.1017/S1368980025101195.

## References

[1] Ministry of Health (2023) Nutrition Survey. https://www.health.govt.nz/statistics-research/surveys/past-surveys/nutrition (accessed 25 November 2024).

[2] Ministry of Health (2003) NZ Food NZ Children: Key Results of the 2002 National Children’s Nutrition Survey. Wellington: Ministry of Health.

[3] Ministry of Health (2011) A Focus on Nutrition: Key Findings of the 2008/09 New Zealand Adult Nutrition Survey. New Zealand: Ministry of Health, Wellington.

[4] Ministry of Health (2022) Scoping Work is Underway to Develop a New Zealand Nutrition Survey. https://www.health.govt.nz/news-media/news-items/scoping-work-underway-develop-new-zealand-nutrition-survey (accessed 18 March 2023).

[5] Parnell WR , Reid J , Wilson NC et al. (2001) Food security: is New Zealand a land of plenty? New Zealand Med J 114, 141–145. 11346164

[6] Ni Mhurchu C , Te Morenga L , Mackay S et al. (2023) New Zealand Nutrition Survey Development: Final Report and Recommendations. Auckland: National Institute for Health Innovation.

[7] Wolcott MD & Lobczowski NG (2021) Using cognitive interviews and think-aloud protocols to understand thought processes. Curr Pharm Teach Learn 13, 181–188. 33454077 10.1016/j.cptl.2020.09.005

[8] Willis GB (2004) Cognitive Interviewing: A Tool for Improving Questionnaire Design. Thousand Oaks, CA: Sage Publications.

[9] Collins D (2003) Pretesting survey instruments: an overview of cognitive methods. Qual Life Res 12, 229–238. 12769135 10.1023/a:1023254226592

[10] Stats NZ (2024) 2023 Census Population Counts (by Ethnic Group, Age, and Māori Descent) and Dwelling Counts. https://www.stats.govt.nz/information-releases/2023-census-population-counts-by-ethnic-group-age-and-maori-descent-and-dwelling-counts/ (accessed 25 November 2024).

[11] Wolfe WS , Frongillo E & Acassano PA (2001) Evaluating brief measures of fruit and vegetable consumption frequency and variety: cognition, interpretation, and other measurement issues. J Am Dietetic Assoc 101, 311–318. 10.1016/S0002-8223(01)00081-5 11269609

[12] Nikolaus CJ , Ellison B & Nickols-Richardson SM (2019) College students’ interpretations of food security questions: results from cognitive interviews. BMC Public Health 19, 1282. 31604466 10.1186/s12889-019-7629-9 PMC6788030

[13] Miller K , Chepp V , Willson S et al. (2014) Cognitive Interviewing Methodology. Hoboken, NJ: John Wiley & Sons.

[14] Gerritsen S , Rosin M , Te Morenga L et al. (2024) Awareness, support, and opinions of healthy food and drink policies: a survey of staff and visitors in New Zealand healthcare organisations. BMC Public Health 24, 2179. 39135033 10.1186/s12889-024-19693-2 PMC11318292

[15] Ministry of Health (2020) Questionnaires and Content Guide 2019/20: New Zealand Health Survey. Wellington: Ministry of Health.

[16] Willis GB (1999) Cognitive Interviewing: A ‘How To’ Guide. Research Triangle Park, NC: Research Triangle Institute.

[17] Centers for Disease Control and Prevention (CDC) (2015) NHANES National Youth Fitness Survey. https://wwwn.cdc.gov/Nchs/Nnyfs/Y_DEMO.htm#DMDFMSIZ (accessed 03 December 2024).

[18] World Health Organization (2024) STEPS Instrument. ∼ https://www.who.int/teams/noncommunicable-diseases/surveillance/systems-tools/steps/instrument#::text=The%20STEPS%20instrument%20is%20comprised,Step%203%20(biochemical%20measures) (accessed 3 December 2024).

[19] Drolet-Labelle V , Bédard A , Lemieux S et al. (2024) Development and cognitive testing of a food frequency questionnaire to assess intake of plant-based protein foods among older adults. Public Health Nutrition 1–28. 10.1017/S1368980024002052 PMC11822641 39431365

[20] Thompson C , Adams J & Vidgen HA (2022) Progressing the development of a food literacy questionnaire using cognitive interviews. Public Health Nutr 25, 1968–1978. 34743775 10.1017/S1368980021004560 PMC9991617

[21] Carbone ET , Campbell MK & Honess-Morreale L (2002) Use of cognitive interview techniques in the development of nutrition surveys and interactive nutrition messages for low-income populations. J Am Dietetic Assoc 102, 690–696. 10.1016/s0002-8223(02)90156-2 12008995

[22] Levin K , Willis GB , Forsyth BH et al. (2009) Using cognitive interviews to evaluate the Spanish-language translation of dietary questionnaire. Surv Res Methods 3, 13–25.

[23] Ashok S , Kim SS , Heidkamp RA et al. (2022) Using cognitive interviewing to bridge the intent-interpretation gap for nutrition coverage survey questions in India. Matern Child Nutr 18, e13248. 34431603 10.1111/mcn.13248 PMC8710093

[24] Khadka S , Sharma N , Yuen-Esco K et al. (2024) Cognitive interviewing to improve infant and young child dietary assessments in the Nepal Demographic and Health Survey. Curr Dev Nutr 9, 104453. 10.1016/j.cdnut.2024.104453 PMC12125683 40454160

[25] Subar AF , Thompson FE , Smith AF et al. (1995) Improving food frequency questionnaires: a qualitative approach using cognitive interviewing. J Am Dietetic Assoc 95, 781–788. 10.1016/s0002-8223(95)00217-0 7797809

[26] Heesch KC , van Uffelen JG , Hill RL et al. (2010) What do IPAQ questions mean to older adults? Lessons from cognitive interviews. Int J Behav Nutr Phys Act 7, 1–13. 20459758 10.1186/1479-5868-7-35 PMC3224924

[27] McClain AC , Johnson CM , DiRado-Owens C et al. (2023) How do Latina/o parents interpret and respond to the US Household Food Security Survey Module? A qualitative cognitive interviewing study. J Acad Nutr Diet 123, S25–S45. 37730305 10.1016/j.jand.2023.07.007 PMC10581700

[28] Calloway EE , Carpenter LR , Gargano T et al. (2022) Development of new measures to assess household nutrition security, and choice in dietary characteristics. Appetite 179, 106288. 36049571 10.1016/j.appet.2022.106288

[29] Byker Shanks C , Calloway EE , Parks CA et al. (2020) Scaling up measurement to confront food insecurity in the USA. Translational Behav Med 10, 1382–1389. 10.1093/tbm/ibaa112 PMC7796720 33277900

[30] Coates J (2013) Build it back better: deconstructing food security for improved measurement and action. Global Food Secur 2, 188–194.

[31] HLPE (2020) *Food Security and Nutrition: Building a Global Narrative Towards 2030. Report by the High Level Panel of Experts on Food Security and Nutrition of the Committee on World Food Security*. Rome: FAO.

[32] Calloway EE , Carpenter LR , Gargano T et al. (2023) New measures to assess the ‘Other’ three pillars of food security–availability, utilization, and stability. Int J Behav Nutr Physical Activity 20, 51. 10.1186/s12966-023-01451-z PMC10134599 37101157

