# Supplementary material for: Testing and adapting dietary habits and food security questions for a national nutrition survey using cognitive interviews and expert consultation
Source: Public Health Nutr. 2025 Oct 6;28(1):e185. doi: 10.1017/S1368980025101195 (PMC12722100; doi:10.1017/S1368980025101195)
Supplement: Follong et al. supplementary material 1 — Follong et al. supplementary material [file S1368980025101195sup001.pdf]

On <u>average</u>, how many <u>servings</u> of fruit do you eat per day? Please include fresh, frozen, canned and stewed fruit.

Do <u>not</u> include fruit juice or dried fruit.

A ‘<u>serving</u>’ of fruit:

1 medium piece of fruit OR 2 small pieces of fruit OR ½ a cup of stewed fruit

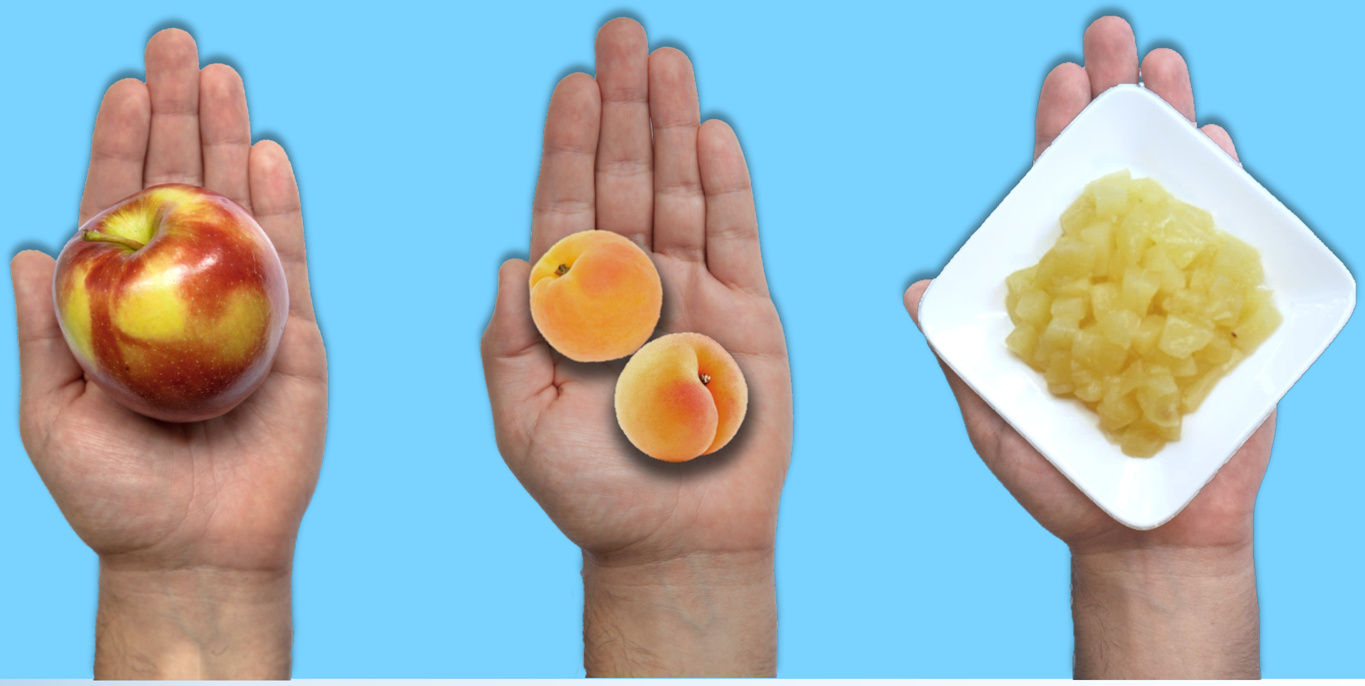

For example: 1 apple + 2 small apricots = 2 servings.

1. I don’t eat fruit
2. Less than 1 serving per day
3. 1 serving per day
4. 2 servings per day
5. 3 servings per day
6. 4 or more servings per day

Do you eat any of the following foods?
Select all that apply.

1 **Red meat**
(e.g. beef, pork, mutton, lamb, goat, venison)

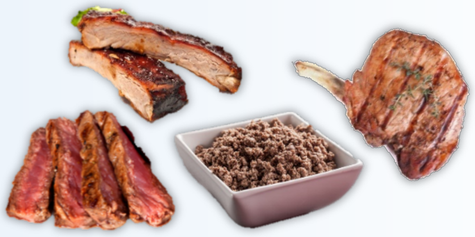

2 **Chicken or poultry**
(e.g. turkey, duck)

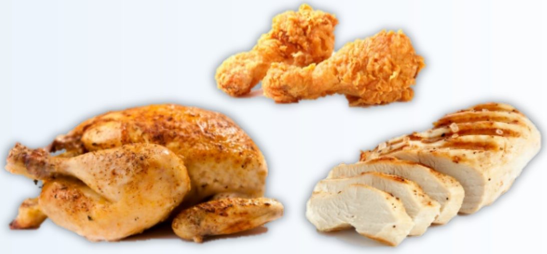

3 **Fish or other seafood**

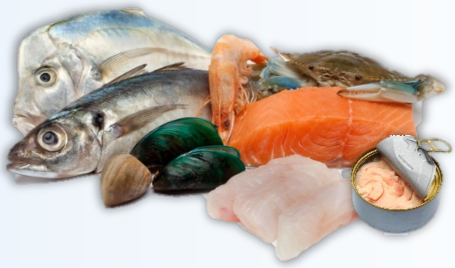

4 **Eggs**

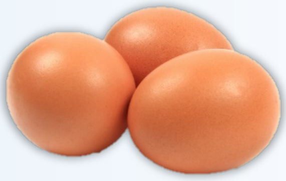

5 **Dairy products**
(e.g. milk, cheese)

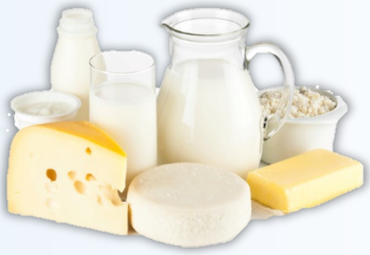

6 **Gluten sources**
(e.g. wheat, barley)

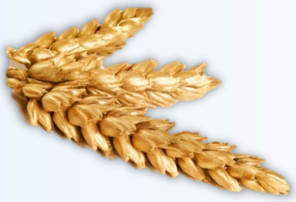

7 **Nuts**

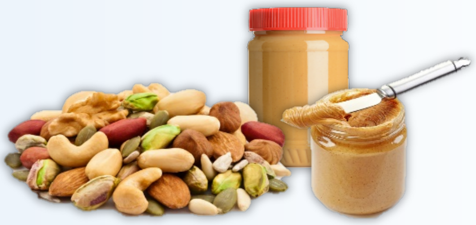

8 **None** of the above
