# Supplementary material for: Testing and adapting dietary habits and food security questions for a national nutrition survey using cognitive interviews and expert consultation
Source: Public Health Nutr. 2025 Oct 6;28(1):e185. doi: 10.1017/S1368980025101195 (PMC12722100; doi:10.1017/S1368980025101195)
Supplement: Follong et al. supplementary material 2 — Follong et al. supplementary material [file S1368980025101195sup002.docx]

Supplementary Table 1 Overview of the original dietary habits and food security questions, questions for cognitive testing, and reasons for inclusion or changes.

| **#** | **Source** | **Original question** | **Question for cognitive testing** | **Reason for inclusion or changes** |
| --- | --- | --- | --- | --- |
| **1** | NZ Dietary Habits Questionnaire | On average, how many servings of fruit do you eat per day? Please include all fresh, frozen, canned and stewed fruit. Do not include fruit juice or dried fruit.  A ‘serving’ = 1 medium piece or 2 small pieces of fruit or ½ cup of stewed fruit.  For example, 1 apple + 2 small apricots = 2 servings.   - I don't eat fruit - Less than 1 serving per day - 1 serving per day - 2 servings per day - 3 servings per day - 4 or more servings per day - Don't know - Refused | The original question was used. | - Question that requires a high cognitive load to answer. - Suspected issues with the terms ‘servings’, ‘average’, and ‘fruit juice’. - Suspected difficulties with the inclusion of the correct foods (e.g., questioned whether fruits added to smoothies would be included in respondent’s answers). |
| **2** | NZ Dietary Habits Questionnaire | On average, how many slices of bread, toast, or bread rolls do you eat per day?  Please don’t include bagels or wraps.   - None - I don't eat bread toast or rolls - Less than one per day - 1-2 per day - 3-4 per day - 5-6 per day - 7 or more per day - Don't know - Refused | On average, how many servings of bread do you eat per day?  A ‘serving’ = 1 slice of bread (40g) or ½ medium bread roll (40g) or ½ naan bread (35g).  Other examples: ½ English muffin or 1 pita bread.   - I don't eat bread - Less than 1 serving per day - 1 serving per day - 2 servings per day - 3 servings per day - 4 servings per day - 5 servings per day - 6 or more servings per day - Don't know - Prefer not to say | - The question will be used to compare against the dietary guidelines and therefore needed to be rewritten to capture the number of servings consumed (similar to the fruit question). - As this question will also be used to monitor iodine and folic acid fortification, bagels and wraps should be included. - Suspected issues with the term ‘servings’. - Suspected difficulties with the inclusion of the correct foods (e.g., questioned what type of foods would be included in respondent’s answers). |
| **3** | NZ Dietary Habits Questionnaire | Do you completely exclude any of the following food groups from your diet?   - Red meat (e.g., beef, pork, mutton, lamb, goat, venison) - Chicken or poultry (e.g., turkey, duck) - Fish or other seafood - Eggs - Dairy products (e.g., milk, cheese) - Gluten sources (e.g., wheat, barley) - Nuts - Don’t know - Refused | Do you eat any of the following foods?  Select all that apply.   - Red meat (e.g., beef, pork, mutton, lamb, goat, venison) - Chicken or poultry (e.g., turkey, duck) - Fish or other seafood - Eggs - Dairy products (e.g., milk, cheese) - Gluten sources (e.g., wheat, barley) - Nuts - Don’t know - Prefer not to say | - Decided to change the wording to what is used in the EPIC Oxford questionnaire as this would be simpler, more positive and less strict than ‘completely exclude’. - Suspected difficulties with answering the question if respondents limit their food intake for specific foods, but do not completely exclude them. |
| **4** | 2002 NZ Children’s Nutrition Survey | Does the person who prepares your meals add salt when they are cooking?   - Yes usually - Yes sometimes - No - Don’t know | How often do you/does the person who prepares your food add salt when you/they are cooking or preparing food?   - Never - Rarely - Sometimes - Regularly - Always - Don’t know - Prefer not to say | - Needed to include salt added by the respondent themselves and by those who do the cooking or preparing of food. - Wanted to find out about salt added while both preparing and cooking food. - Answer options needed to align with another question on salt intake not part of the cognitive testing (i.e., How often do you add salt to your food after it has been cooked or prepared?) - Suspected difficulties with the inclusion of all meals and snacks consumed throughout the day (e.g., questioned which meals or snacks would be included in respondent’s answers). - Suspected difficulties with the inclusion of all meals and snacks consumed throughout the day (e.g., questioned whether respondents know how often someone else adds salt to their food). |
| **5** | Growing Up in New Zealand (NZ cohort study) | In the past 12 months, have you used any of the following food programmes?   - Breakfast programme - Fruit in schools - Milk in schools - Free and Healthy School Lunch programme (Government funded) - Charitable food programme (e.g., KidsCan, Eat My Lunch) | In the past 12 months, have you used any of the following food programmes?  Please select all that apply.   - Breakfast programme - Fruit in schools - Milk in schools - Free and Healthy School Lunch programme (e.g., Ka Ora, Ka Ako) - Charitable food programme (e.g., KidsCan, Eat My Lunch) - Other, please specify (leave blank if unknown) - I don’t receive food and/or drinks from food programmes - Don’t know - Prefer not to say | - Provided clearer examples and instructions. - Added an option to report other school food programmes not listed or to indicate that no food/drink was received in the past 12 months. - Suspected difficulties with the inclusion of the correct programmes (e.g., questioned whether non-school-based programmes would be included in respondent’s answers). |
| **6^** |  | New question. | In the past 12 months, has your household eaten foods from any of the following sources?  Include both foods that your household has gathered/collected and those that were given to you.  Please select all that apply.   - Hunting - Fishing/diving - Home kill - Foraging (fruit, vegetables, nuts, herbs, mushrooms) - Your (community) garden - Collecting fresh eggs - Milking cows/sheep/goats - Other, please specify - No, I buy all my food - Don’t know - Prefer not to say | - Included as knowing where people get their food from was considered important. Data on food procurement cannot be obtained through a 24-hour dietary recall and was therefore added as a question in the national nutrition survey. - The purpose of the question was to understand how often people consume foods from non-monetary sources. - Wanted to find out if respondents would be able to answer this question for their household. - Wanted to find out if there were any sources missing that would be highlighted by the cognitive testing participants. |
|  |  |  | *Followed by a frequency input for any selected foods.*  How often does your household eat foods from [food source]?   - Most days - Weekly - Monthly - 3-4 times per year - 1-2 times per year - Don’t know - Prefer not to say | - Wanted to find out how respondents would recall the frequency of consumption of food from these sources within a 12-month time frame (e.g., some foods may be sourced only seasonally). |
| **7** | US NHANES | Have you used or taken any vitamins, minerals, herbals or other dietary supplements in the past 30 days? Include prescription and non-prescription supplements.   - Yes - No - Refused - Don't know | Have you taken any dietary supplements in the past month?  Include any prescription and over the counter supplements.   - Yes - No - Don't know - Prefer not to say | - Question was simplified by removing words that were perceived as unnecessary or using lay terms. - Suspected issues with the terms ‘dietary supplements’, ‘prescription’, ‘over the counter’. |
| **8^** | NZ Food Security Questionnaire | *We are interested in whether you run out of basics, like bread, potatoes, etc. because you do not have enough money. We are NOT referring to treats or special foods.*  Food runs out in my/our household due to a lack of money.  How often has this been true for you (or your household) over the past year?   - Often - Sometimes - Never - Don’t know - Refused | The original question was used. | - Question that was believed to no longer capture food insecurity as experienced in the modern context given the changes in the food environment. - Suspected issues with the examples used for ‘basic’ foods and believed these needed to be more culturally diverse. |
| **9^** | NZ Food Security Questionnaire | *Some people rely on support and assistance from others for supplying their regular food and we are interested in finding out how many people fall into this group.*  I/we rely on others to provide food and/or money for food, for my/our household, when I/we don’t have enough money.  How often has this been true for you (or your household) over the past year?   - Often - Sometimes - Never - Don’t know - Refused | The original question was used. | - Wanted to understand how ‘others’ was interpreted and if this needed to be clarified. - Wanted to find out what respondents consider food support or assistance. |
| **10^** | NZ Food Security Questionnaire | *Also, some people have to rely on other sources of help such as food grants or food banks.*  I/we make use of special food grants or food banks when I/we do not have enough money for food.  How often has this been true for you (or your household) over the past year?   - Often - Sometimes - Never - Don’t know - Refused | The original question was used. | - Wanted to find out how the terms ‘food grants’ and ‘food banks’ was interpreted and if this needed to be clarified. - Wanted to find out if respondents included what other sources of food assistance in their answers to this question. |
| **11^** |  | New question based on NZ Food Security Questionnaire. | *Some people have to rely on other sources of help such as their church, their marae or other community organisations.*  I/we receive support from a church, marae or other community organisation when I/we do not have enough money for food.  How often has this been true for you (or your household) over the past year?   - Often - Sometimes - Never - Don’t know - Prefer not to say | - Decided to explore whether participants include those less formal food assistance sources by including a new question specifically asking about these sources. - The landscape of food assistance has changed over time and if alternatives are not included then will lead to underestimating and underrepresenting the role churches, marae, and other community food assistance programmes. |
| **12^** |  | New question. | Are your current food preparation and food storage facilities adequate to prepare food for your household?   - Yes - No - Don’t know - Prefer not to say | - Included to find out if there may be an association between the lack of food preparation and storage facilities and peoples’ food intake. - Suspected issues with the term ‘adequate’. - Suspected difficulties with the inclusion of preparation and storage facilities (e.g., questioned whether participants would include all possible facilities in their answer). |

^^^Questions were only asked to adult participants.
